# CRISPR-based functional genomic screening in neurodegeneration: mechanistic insights into AD, PD, and ALS

**DOI:** 10.3389/fnagi.2026.1831375

**Published:** 2026-09-08

**Authors:** Feng Xue, Lili Bao, Anda He, Sarentuya Liang

**Affiliations:** 1 Department of Surgery, Friedrich-Alexander-Universität Erlangen-Nürnberg (FAU) and Universitätsklinikum Erlangen, Erlangen, Germany; 2 Department of Medicine 1, Friedrich-Alexander-Universität Erlangen-Nürnberg (FAU) and Universitätsklinikum Erlangen, Erlangen, Germany; 3 Inner Mongolia Autonomous Region Key Laboratory of Medicine and Health: Pharmacology of Mongolian Medicine, Affiliated Hospital of Inner Mongolia Minzu University, Tongliao, Inner Mongolia Autonomous Region, China; 4 The Second Department of Encephalopathy, Affiliated Hospital of Inner Mongolia Minzu University, Tongliao, Inner Mongolia Autonomous Region, China

**Keywords:** Alzheimer's disease, amyotrophic lateral sclerosis, CRISPR-based screening, neurodegeneration, Parkinson's disease

## Abstract

Neurodegenerative diseases, including Alzheimer's disease (AD), Parkinson's disease (PD), and amyotrophic lateral sclerosis (ALS), are characterized by pronounced clinical and molecular heterogeneity, as well as highly interconnected pathogenic pathways. This biological complexity has long hindered efforts to systematically define disease mechanisms and to develop effective, targeted therapies. In recent years, clustered regularly interspaced short palindromic repeats (CRISPR) based functional genomic screening technologies have emerged as powerful tools for large-scale genetic perturbation in cellular, organoid, and *in vivo* models, enabling unbiased interrogation of disease relevant genetic networks and the identification of potential therapeutic targets. In this review, we summarize CRISPR knockout, CRISPR interference, CRISPR activation, and *in vivo* screening studies in AD, PD, and ALS, with emphasis on pathological phenotypes, experimental models, cell types, validation strategies, and evidence strength. In AD, these screens have identified regulators of amyloid-β (Aβ) production, Tau homeostasis and propagation, microglial states, neuronal aging, and stress responses. In PD, they have provided insights into α-synuclein (α-syn) homeostasis, mitochondrial quality control, lysosomal trafficking, and transplanted dopaminergic neuron survival. In ALS, they have identified modifiers of C9orf72-associated toxicity, repeat-associated non-AUG translation, TAR DNA-binding protein 43 (TDP-43) inclusion formation, and ATXN2 homeostasis. Cross-disease comparison indicates recurring involvement of proteostasis, endolysosomal function, mitochondrial regulation, and cellular stress responses, although individual screening hits show limited overlap and remain strongly influenced by experimental context. Overall, CRISPR-based screening provides a useful framework for identifying candidate disease modifiers, but further validation across complementary human-relevant and *in vivo* models is required before therapeutic translation.

## 1 Introduction

Neurodegenerative diseases refer to a group of disorders that primarily affect neurons, the fundamental units of the nervous system. These diseases are characterized by the gradual loss of neuronal function and structure, leading to progressive decline in cognitive, motor, and emotional abilities (Gadhave et al., 2024). Common neurodegenerative diseases include Alzheimer's disease (AD), Parkinson's disease (PD), Huntington's disease (HD), and amyotrophic lateral sclerosis (ALS) (Wang et al., 2024; Su et al., 2025). With the accelerating aging of the global population, the incidence and socioeconomic burden of neurodegenerative diseases have increased significantly, becoming one of the major public health challenges worldwide (Wang et al., 2024; Su et al., 2025). Although the clinical symptoms and pathological features of these diseases have been relatively well described, their complex molecular mechanisms remain poorly understood, involving multiple interacting factors such as abnormal protein aggregation, mitochondrial dysfunction, oxidative stress, and neuroinflammation (Cullinane et al., 2024; Wilson et al., 2023; Robinson et al., 2023). Neurodegeneration is also influenced by interactions among multiple cell types. In addition to vulnerable neuronal populations, microglia, astrocytes, oligodendrocytes, and vascular associated cells may contribute to disease progression through their effects on inflammatory signaling, pathological protein clearance, metabolic support, synaptic function, and tissue homeostasis (Gao et al., 2023; Liu et al., 2023; Kempuraj et al., 2024). These cell type specific responses and intercellular interactions add another level of complexity and may influence the genetic modifiers identified in different experimental models. These complexities limit the effectiveness of traditional research approaches; for instance, conventional candidate gene studies and small-scale mutation models may be insufficient to systematically characterize these complex regulatory networks.

Over the past decade, driven by rapid progress in genomics and molecular technologies, the clustered regularly interspaced short palindromic repeats (CRISPR) system has become a powerful tool in functional genomics research due to its high specificity and programmable gene editing capability (Bock et al., 2022; Sharma et al., 2021). This technology not only enables precise editing of specific genes but also allows the construction of libraries containing tens of thousands of single guide RNAs (sgRNAs) combined with high-throughput screening strategies (Wang et al., 2014; Chen et al., 2025). Researchers can systematically evaluate gene functions in neural cells or animal models to identify candidate genes and regulatory pathways associated with neurodegenerative diseases, providing opportunities to investigate disease mechanisms and prioritize potential therapeutic targets (Rahimi et al., 2024; Lu et al., 2021; Wilson and Metzakopian, 2021).

**Figure 1:**
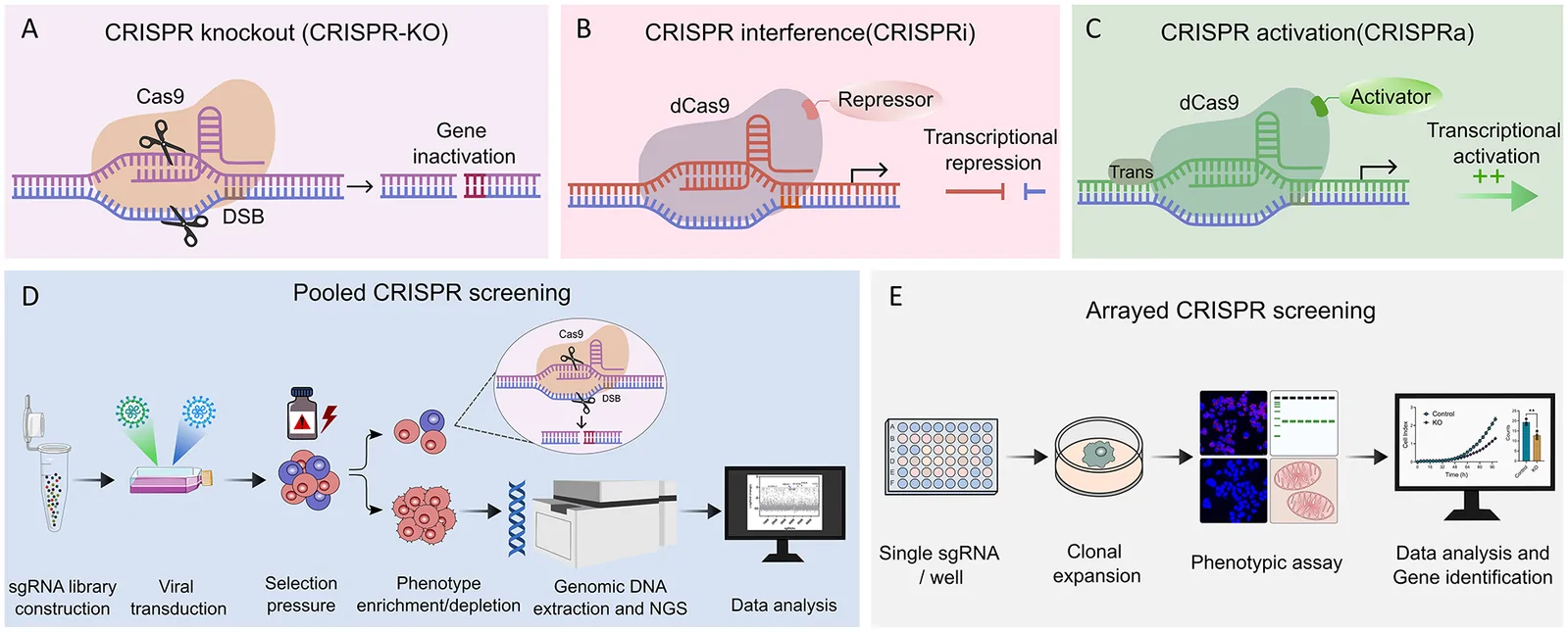
CRISPR-based gene perturbation and screening strategies. **(A)** CRISPR KO: Wild-type Cas9 nuclease directs DNA cleavage at the coding region of a target gene guided by a sgRNA. The resulting double-strand breaks (DSB) undergo error-prone repair via the non-homologous end joining (NHEJ) pathway, leading to frameshift mutations that disrupt gene function. **(B)** CRISPRi: A nuclease-deactivated Cas9 (dCas9) mediates the recruitment of a transcriptional repressor (e.g., KRAB) to the transcription start site (TSS), thereby silencing gene expression. **(C)** CRISPRa: A dCas9 mediates the recruitment of a transcriptional activator (e.g., VP64) to the TSS, inducing or enhancing gene transcription. **(D)** Pooled screening: Cells are transduced with a library of sgRNAs. After phenotypic selection, sgRNA abundance is quantified via NGS to identify hits. **(E)** Arrayed screening: Individual sgRNAs are spatially segregated in multi-well plates (one-gene-per-well), allowing direct evaluation of complex phenotypes (e.g., morphology) via high-content assays.

CRISPR/Cas9 technology originated from the adaptive immune system of bacteria and has evolved in recent years into a powerful gene editing tool (Pacesa et al., 2024). Its fundamental mechanism involves the Cas9 nuclease recognizing and cleaving specific DNA sequences under the guidance of a sgRNA, thereby enabling gene knockout or repair (Pacesa et al., 2024; Leonova and Gainetdinov, 2020). In functional genomics research, CRISPR/Cas9 facilitates high-throughput screening using genome-wide knockout libraries (CRISPR-KO) as well as the development of CRISPR interference (CRISPRi) and CRISPR activation (CRISPRa) approaches (Figures 1A–C). These utilize inactivated Cas9 (dCas9) fused with transcription activators or repressors to achieve reversible regulation of gene expression levels (Kampmann, 2018). This flexibility confers significant advantages for studying gene-dose-sensitive cell types such as neurons. Applications of CRISPR screening in disease research primarily focus on elucidating pathogenic mechanisms, validating risk gene functions, and identifying potential therapeutic targets.

According to the various strategies employed in the study of gene function and the design of experiments, CRISPR/Cas9 screening can be broadly divided into two main categories (Bock et al., 2022). The first approach involves virus vector-based large-scale gene knockout screening (CRISPR knockout screening) (Figure 1D). In this approach, different sgRNAs are transduced into a single cell population. By combining phenotypic selection with high-throughput sequencing, key genes that influence a specific phenotype can be identified. The underlying mechanism of this method involves the CRISPR/Cas9 system inducing double-strand breaks (DSBs) at specific genomic loci. During repair via non-homologous end joining (NHEJ), insertions or deletions (indels) are introduced, leading to frameshift mutations and loss of gene function (Kerek et al., 2021). In experimental applications, researchers first generate a sgRNA library containing thousands of distinct sgRNAs, which is subsequently delivered into a cell population, ensuring that each cell harbors a targeted knockout of a single gene. The cell population is subsequently subjected to defined selection pressures, such as exposure to pharmacological agents, environmental stressors, or prolonged culture conditions, allowing for the enrichment or depletion of cells harboring knockouts in genes that influence cellular survival, proliferation, or other functional characteristics. Finally, high-throughput sequencing is performed to quantify sgRNA abundance, and comparison between experimental and control groups enables the identification of genes essential for cell viability, drug response, or functional performance under specific conditions, thereby facilitating systematic functional genomic screening (Bock et al., 2022; Park et al., 2025). The second category is arrayed CRISPR screening (Figure 1E), which is well-suited for detailed phenotypic analyses. This strategy involves the independent transduction of individual sgRNAs or small sets of sgRNAs into separate wells, for example in multi-well plates. Each well of the plate is designed to target a specific gene, thereby enabling individual phenotypic assessment of each editing event (Yin et al., 2025). This approach is particularly valuable for the analysis of complex phenotypes, including neuronal morphology changes, cell polarity, mitochondrial dynamics, synapse formation and calcium signaling activity (Wilson and Metzakopian, 2021; Yin et al., 2025).

Previous disease focused reviews have summarized the broader applications of CRISPR/Cas9 technology in AD, PD, and ALS, including disease modeling, therapeutic genome editing, stem cell-based approaches, delivery strategies, and translational challenges (Khan et al., 2025; Rahman et al., 2022; Shi et al., 2023). For example, Khan et al. reviewed CRISPR/Cas9-based therapeutic approaches in AD, whereas other reviews have examined its applications in PD and ALS disease modeling and therapeutic development (Khan et al., 2025; Rahman et al., 2022; Shi et al., 2023). In contrast, the present review focuses specifically on CRISPR-based functional screening studies in AD, PD, and ALS, with emphasis on the pathological phenotypes investigated, experimental models and cell types, screening strategies, validation approaches, strength of evidence, and translational potential.

AD, PD, and ALS represent distinct clinical and pathological forms of neurodegeneration and involve different disease associated proteins, vulnerable cell types, and experimental phenotypes, providing a useful framework for comparative analysis (Kampmann, 2020; Sanchez et al., 2021). Among the studies included in this review, a broader and more diverse range of relevant screening studies was identified for these three diseases than for other neurodegenerative disorders, allowing comparisons across different screening strategies and disease contexts. HD and other neurodegenerative diseases were not included in the main comparative analysis because fewer relevant screening studies were identified within the defined scope of this review. This scope does not imply that these disorders are less important.

The present review summarizes and compares the pathological phenotypes, screening strategies, experimental models, cellular contexts, and validation approaches used in studies of AD, PD, and ALS. We further integrate the reported findings, discuss the limitations, reproducibility, and translational potential of current screening approaches, and highlight future directions in this rapidly evolving field.

## 2 Applications of CRISPR-based screening in AD

AD is the most common neurodegenerative disorder worldwide and the leading cause of dementia, accounting for approximately 60–70% of all dementia cases (Frisoni et al., 2025a). Global epidemiological estimates from 2021 indicate that nearly 57 million people are affected by some form of dementia. With the accelerating aging of the population, the number of individuals affected by AD continues to rise and is projected to nearly triple by 2050 (Frisoni et al., 2025a). Clinically, AD is characterized by chronic, progressive impairments in memory, language, visuospatial orientation, and executive function, often accompanied by behavioral disturbances (Frisoni et al., 2025a; Fox et al., 2025). From a pathological and biomarker perspective, AD is commonly described using the amyloid/tau/neurodegeneration AT(N) framework. In this framework, “A” refers to biomarkers of amyloid-β (Aβ) deposition, “T” refers to biomarkers of pathological tau, and “(N)” refers to biomarkers of neurodegeneration or neuronal injury. Aβ accumulation is often detectable early in the disease course, whereas tau pathology and neurodegenerative changes generally become more prominent as the disease progresses. Histopathologically, these processes are reflected by extracellular amyloid plaques, intracellular neurofibrillary tangles and neuropil threads, as well as synaptic dysfunction and neuronal loss, which collectively contribute to progressive cognitive impairment (Frisoni et al., 2025a; Fox et al., 2025). These irreversible neurodegenerative changes drive the molecular basis of the cognitive and behavioral deficits observed in AD. Throughout disease evolution, patients gradually lose the capacity to perform basic activities of daily living, exhibit significantly reduced life expectancy, and generate a considerable long-term caregiving and economic burden for both families and society (Frisoni et al., 2025a; Fox et al., 2025; Frisoni et al., 2025b).

AD can be classified into familial (FAD) and sporadic (SAD) forms (Andrade-Guerrero et al., 2023). FAD, accounting for approximately 1–5% of all cases, predominantly exhibits an autosomal dominant inheritance pattern and typically presents before 65 years of age. It is mainly associated with pathogenic mutations in *PSEN1, PSEN2*, and *APP*. In comparison, SAD, which represents the majority of cases (~95%), usually develops after the age of 65 and is influenced by a combination of genetic and environmental factors such as aging, sex, depression, traumatic brain injury, lifestyle, and metabolic syndrome, with aging being the predominant risk factor (Andrade-Guerrero et al., 2023; Krishnamurthy et al., 2025). Genetic studies have revealed that the etiology of AD is highly complex, involving both rare, highly penetrant mutations and numerous common variants with modest effects. The *APOE* ε4 allele remains the strongest genetic risk factor identified to date, while large-scale genome-wide association studies (GWAS) have uncovered over 70 additional susceptibility loci, including *TREM2, ABCA7, SORL1*, and *BIN1*, implicating diverse pathways related to immune response, lipid metabolism, and proteostasis (Reitz et al., 2023; Andrews et al., 2023; De Deyn and Sleegers, 2025). However, the functional roles and interactions of most risk genes remain poorly understood, limiting the translation of genetic discoveries into therapeutic strategies. CRISPR/Cas9-based functional screening may help characterize candidate risk genes and identify the regulatory pathways involved in AD.

### 2.1 CRISPR screening identifies regulators of Aβ production

**Figure 2:**
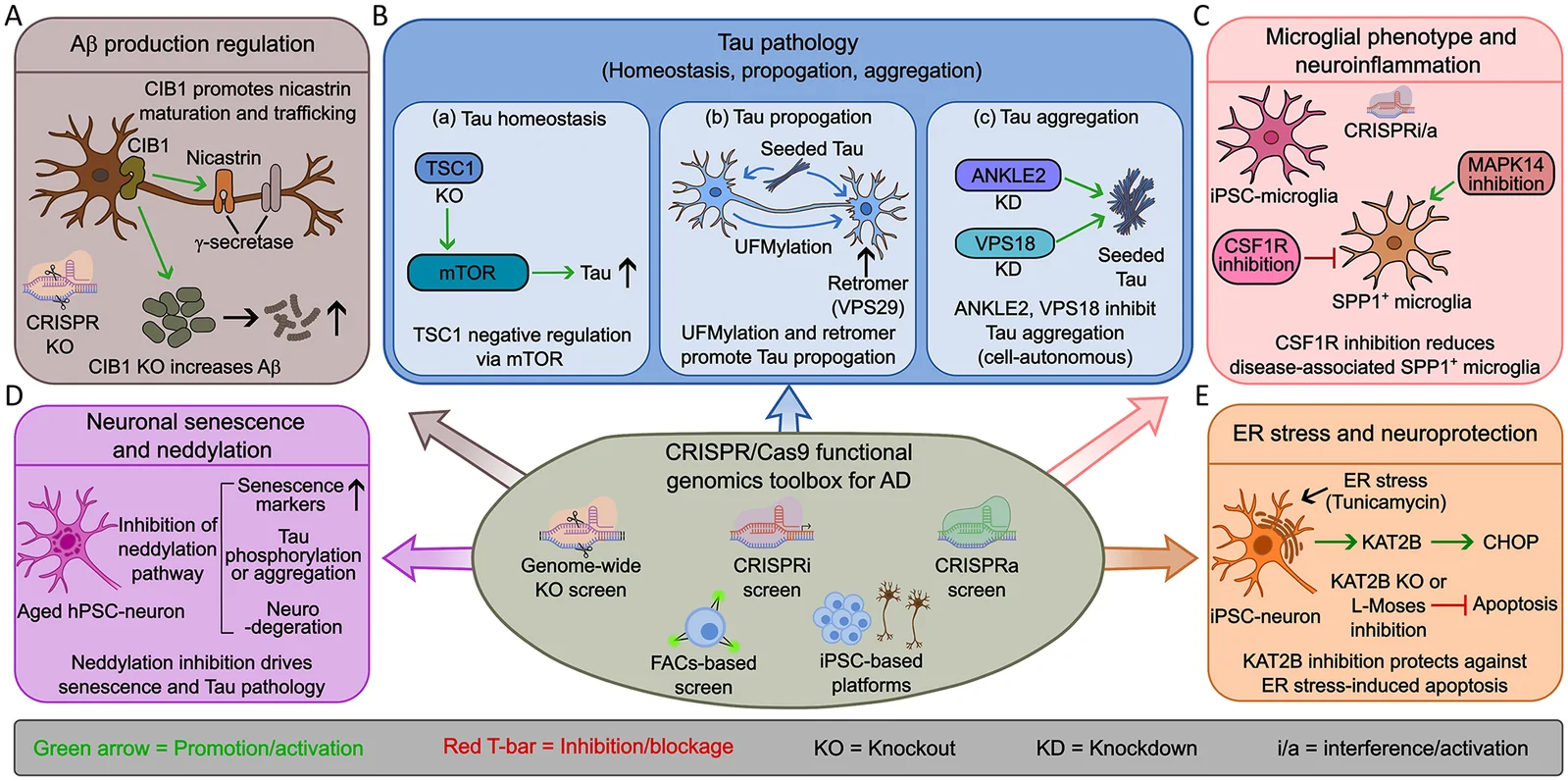
CRISPR functional genomics reveals multiple pathological mechanisms in AD. **(A)** Aβ production regulation. CIB1 acts as a negative regulator of Aβ production by facilitating cell-surface trafficking of mature Nicastrin (within the γ-secretase complex); its loss elevates Aβ levels. **(B)** Tau Pathology. **(a)** Homeostasis: *TSC1* knockout increases tau levels through mTOR signaling. **(b)** Propagation: The UFMylation cascade positively regulates tau propagation; its knockdown inhibits seeded spreading in a 4R tauopathy model. **(c)** Aggregation: *ANKLE2* and *VPS18* suppress intracellular tau aggregation; their knockdown enhances pathology post-endocytosis. **(C)** Microglial phenotype and neuroinflammation. CROP-seq reveals bidirectional regulation of SPP1^+^ disease-associated microglia: inhibition of CSF1R depletes this population, whereas MAPK14 inhibition promotes its expansion. **(D)** Neuronal senescence and neddylation. Integration of senescence markers into screening highlights the neddylation pathway as a guardian of homeostasis; its loss exacerbates tau pathology in *APP*^swe/swe^ neurons. **(E)** ER stress and neuroprotection. Screening the druggable genome identifies *KAT2B* as a regulator of the unfolded protein response (UPR). Pharmacological inhibition of KAT2B (L-Moses) attenuates CHOP activation and prevents neuronal death in AD models.

In the context of Aβ metabolism, a genome-wide CRISPR/Cas9 knockout screen in mouse neuroblastoma Neuro2a (N2a) cells identified calcium and integrin-binding protein 1 (CIB1) as a candidate negative regulator of Aβ production (Figure 2A) (Chiu et al., 2020). Loss of CIB1 significantly elevated Aβ levels, not through direct effects on γ-secretase enzymatic activity, but by affecting the maturation and cell-surface trafficking of Nicastrin, a key component of the γ-secretase complex (Chiu et al., 2020). Notably, CIB1 expression was reduced in neurons from patients with early-stage AD, suggesting a potential neuroprotective role. This study identified a previously unrecognized mechanism of Aβ regulation in N2a cells and nominated CIB1 as a candidate modifier for further validation in human neuronal models (Chiu et al., 2020).

### 2.2 Genetic elucidation of tau pathology using CRISPR-based screening

To systematically map the genetic landscape of endogenous tau regulation, a fluorescence-activated cell sorting (FACS)-based genome-wide CRISPR/Cas9 knockout screen was conducted in SH-SY5Y human neuroblastoma cells, with selected hits subsequently tested in Ngn2-induced human excitatory glutamatergic neurons (Sanchez et al., 2021). The primary screen identified 481 candidate genes, of which 266 knockouts decreased tau levels, while 215 increased them. A secondary validation confirmed 93 tau-lowering and 62 tau-elevating genes, with a limited subset, including *TSC1*, validated in human neurons. The study highlighted chromatin-modifying enzymes, the neddylation, ubiquitin-proteasome system (UPS), and the mechanistic target of rapamycin (mTOR) signaling pathway as key regulators of tau homeostasis (Sanchez et al., 2021). In particular, loss of *TSC1* significantly elevated tau levels, supporting its role as a negative regulator of tau abundance (Figure 2B (a)). Several UPS-related genes, including *UBE2A, UBE2H, UBE4B, FBXW7, FBXO11*, and *CUL5*, were also implicated, suggesting that these post-translational regulatory processes influence neuronal tau levels (Sanchez et al., 2021). However, the limited validation of selected candidates in Ngn2-induced neurons indicates that the effects identified in SH-SY5Y cells were strongly influenced by cellular context. In addition, the induced neurons did not fully reproduce the tau isoform profile of the adult human brain.

Addressing the limitation that standard induced pluripotent stem cell (iPSC)-derived neurons often lack sufficient 4R tau expression, recent work used human pluripotent stem cell-derived neurons expressing 4R-P301S tau to model seeded tau aggregation and intercellular propagation (Parra Bravo et al., 2024). A targeted CRISPRi screen covering 1,073 genes identified more than 500 candidate modifiers of tau propagation. Among them, the retromer complex component VPS29 and the UFMylation cascade (*UFM1, UBA5, UFC1, UFL1*, and *DDRGK1*) emerged as major positive regulators (Figure 2B (b)) (Parra Bravo et al., 2024). Functional validation showed that suppression of UFMylation reduced seeded tau spread, while the pathway was aberrantly activated in neurofibrillary tangle-positive neurons from AD and progressive supranuclear palsy (PSP) brains (Parra Bravo et al., 2024). These findings support the view that seeded tau propagation can be modified through defined genetic pathways and nominate UFMylation for further mechanistic and therapeutic investigation in tauopathy models.

Building on investigations of tau propagation, cell-autonomous modulators of tau aggregation were investigated using a genome-wide CRISPRi screen in HEK293-derived tau biosensor cells. Using a Foerster resonance energy transfer (FRET)-based detection system, the study screened for genes that regulate seeded aggregation induced by exosome-associated or free tau species (Polanco et al., 2023). Five candidate genes, including *ANKLE2, BANF1, NUSAP1, EIF1AD*, and *VPS18*, were identified as suppressors of tau aggregation (Figure 2B(c)), as their knockdown enhanced tau seeding by both pathogenic forms (Polanco et al., 2023). These genes did not affect tau uptake, suggesting that they regulate events occurring after cellular uptake rather than tau internalization itself. Importantly, *VPS18, NUSAP1*, and *EIF1AD* were found to be downregulated in postmortem AD brains, supporting their disease relevance (Polanco et al., 2023). The study therefore identified candidate post-uptake regulators of seeded tau aggregation in an engineered biosensor system.

### 2.3 Microglial regulation revealed by CRISPR functional genomics

Given emerging genetic evidence positioning microglia as central drivers of AD pathogenesis, a CRISPRi/a-based functional genomics platform was established in human iPSC-derived microglia to systematically identify key regulators of microglial phenotypes (Efthymiou and Goate, 2017). To overcome the technical limitations of conventional differentiation protocols, the authors developed an 8-day rapid reprogramming strategy by inducing six microglia-related transcription factors (Dräger et al., 2022). This approach generated scalable human microglial cells and achieved stable integration of both CRISPRi and CRISPRa systems. Using a pooled sgRNA library targeting the “druggable genome”, they performed three large-scale screens and identified genes modulating microglial survival/proliferation, inflammatory activation, and phagocytic function. At the single cell level, the study further revealed that the induced microglia spontaneously adopt multiple transcriptional states, including an osteopontin (SPP1^+^) disease-associated state that is highly enriched in AD brains (Dräger et al., 2022). To investigate the genetic circuitry controlling these states, the study employed CRISPR droplet sequencing (CROP-seq), a method integrating pooled CRISPR screening with single-cell transcriptomic readouts (Dräger et al., 2022). The analysis demonstrated that inhibition of *CSF1R* markedly reduces the proportion of SPP1^+^ microglia, whereas *MAPK14* inhibition promots expansion of this state (Figure 2C). Pharmacological validation confirmed that low-dose CSF1R inhibition with PLX3397 selectively depletes SPP1^+^ microglia, while MAPK14 inhibition increases their abundance (Dräger et al., 2022). These findings provide model-specific evidence that microglial transcriptional states can be altered through genetic and pharmacological perturbation in transcription factor-induced human microglia.

### 2.4 Aging-associated pathways identified by CRISPR screening

While traditional screens have prioritized pathways linked to Aβ metabolism, tau stability, or synaptic function, recent efforts have integrated cellular senescence to address the age-dependent nature of AD. In 2024, a genome-wide CRISPR knockout screen was conducted in human pluripotent stem cell (hPSC)-derived cortical neurons, with neuronal aging as a biological variable (Saurat et al., 2024). The neddylation (NEDD8 conjugation) pathway was identified as a central regulator of neuronal aging and AD-like neurodegeneration (Figure 2D) (Saurat et al., 2024). Inhibition of neddylation enhanced cellular senescence markers, promoted tau hyperphosphorylation and aggregation in *APP*^*swe*/*swe*^ neurons, and induced selective neuronal loss across multiple neurodegenerative models (Saurat et al., 2024). These findings support a role for neddylation in maintaining neuronal homeostasis and resistance to aging-associated stress. They also illustrate how induced-aging paradigms can be combined with CRISPR screening to investigate interactions between cellular aging and genetic vulnerability.

### 2.5 CRISPR screens targeting proteostasis and ER stress

To investigate genetic modifiers of protein misfolding-related stress, a CRISPR/Cas9 knockout screen targeting the druggable genome was performed in human iPSC-derived cortical neurons exposed to tunicamycin (Pavlou et al., 2023). This approach aimed to identify modulators of endoplasmic reticulum (ER) stress-induced neuronal death. Combined pooled and arrayed validation screens identified 13 neuroprotective candidates, with the lysine acetyltransferase *KAT2B* emerging as a key regulator (Pavlou et al., 2023). Both genetic ablation of *KAT2B* and pharmacological inhibition using L-Moses significantly mitigated the activation of the pro-apoptotic factor C/EBP-homologous protein (CHOP, also known as DDIT3) and subsequent apoptosis (Figure 2E) (Pavlou et al., 2023). These findings support a role for KAT2B-mediated acetylation in acute tunicamycin-induced ER stress and provide an example of a screening hit followed by pharmacological validation in human cortical neurons.

Collectively, these studies identify candidate regulators of Aβ production, tau homeostasis and propagation, microglial states, aging-related neuronal vulnerability, and ER stress responses. The strength and disease relevance of these findings vary according to the cell type, experimental model, perturbation strategy, and phenotypic readout used. Further validation across independent human-relevant and *in vivo* systems will be important for determining which candidates have broader relevance to AD pathogenesis and therapeutic development (Table 1).

**Table 1:** Summary of CRISPR-based functional genomic screens in AD.

Model	Screening type	sgRNA library	Key findings	Reference
Murine neuroblastoma Neuro2a (N2a) cells	CRISPR/Cas9 knockout pooled screen	pooled library: 87,897 sgRNAs targeting 19,150 genes	Identified *CIB1* as a negative regulator of Aβ production; *CIB1* deletion increases Aβ levels by altering γ-secretase complex maturation and cell-surface localization without affecting enzymatic activity.	Chiu et al., 2020
SH-SY5Y neuroblastoma cells	CRISPR/Cas9 knockout pooled screen	Genome-wide library: ~90,000 sgRNAs, 5 sgRNAs per gene, covering 18,360 genes	Identified genes and pathways regulating endogenous tau, including chromatin modifiers, neddylation/ubiquitin, and mTOR components.	Sanchez et al., 2021
Human iPSC-derived 4R-P301S tauopathy neurons (dCas9-KRAB CRISPRi line)	Pooled CRISPRi screen	Custom CRISPRi lentiviral library: targeting 1,073 tau-pathobiology genes, 5 sgRNAs per gene + 250 non-targeting controls (NTC) sgRNAs	UFMylation pathway promotes seeded tau aggregation and propagation in human neurons.	Parra Bravo et al., 2024
BSKRAB tau biosensor cells + rTg4510 sarkosyl-insoluble tau seeds	CRISPRi pooled functional screen with FACS-based sorting (FRET^+^ vs. FRET^−^ for tau aggregation)	Genome-wide Dolcetto CRISPRi sgRNA library: 57,050 sgRNAs targeting ~18,000 genes	Identified *ANKLE2, BANF1, NUSAP1, EIF1AD*, and *VPS18* as novel regulators of tau pathology induced by both exosome-like extracellular vesicles and free tau seeds.	Polanco et al., 2023
Human iPSC-derived iTF-Microglia	Pooled CRISPRi and CRISPRa screens for survival, activation (CD38 FACS), phagocytosis (pHrodo-synaptosomes FACS), plus CROP-seq state mapping	Druggable genome library: 2,325 genes, 5 sgRNAs per gene, 500 NTC sgRNAs	Genes controlling survival/activation/phagocytosis identified; microglial states mirroring human brain delineated; SPP1^+^ disease-associated state selectively depleted by *CSF1R* inhibition.	Dräger et al., 2022
hPSC-derived cortical neurons	CRISPR/Cas9 knockout pooled screen	Brunello human CRISPR knockout pooled library: 76,441 sgRNAs for 19,114 genes, 1,000 NTC sgRNAs	Identified neddylation pathway as a key regulator of neuronal aging and AD-related neurodegeneration; inhibition of neddylation aggravates tau pathology and neuronal loss, suggesting it as a potential therapeutic target.	Saurat et al., 2024
Human iPSC-derived cortical-like neurons	Druggable genome CRISPR/Cas9 screen + validation array	Druggable genome library: 26,306 sgRNAs targeting 4,401 genes, 1,000 NTC sgRNAs	Identified 13 neuroprotective knockouts under ER stress; *KAT2B* knockout or inhibition by L-Moses reduced CHOP expression and neuronal death.	Pavlou et al., 2023

## 3 Applications of CRISPR-based screening in PD

PD is the second most common neurodegenerative disorder after AD. Its prevalence has continued to rise worldwide, creating an increasing public health burden (Ben-Shlomo et al., 2024). Epidemiological evidence shows that the risk of PD increases with age and is further amplified by population aging. Although PD primarily affects middle-aged and older individuals, with a typical age at onset of approximately 60 years, early-onset cases are being reported more frequently (Ben-Shlomo et al., 2024; Deliz et al., 2024). As a chronic and progressive disorder, PD leads to persistent motor dysfunction, cognitive decline, and multisystem involvement, collectively reducing patients' quality of life and placing long-term demands on healthcare and caregiving systems (Su et al., 2025; Ben-Shlomo et al., 2024; Deliz et al., 2024).

The etiology and pathogenesis of PD are multifactorial and remain incompletely understood. The defining pathological feature of PD is the misfolding, aggregation, and accumulation of α-synuclein (α-syn), leading to the formation of Lewy bodies and Lewy neurites that contribute to neuronal dysfunction and eventual cell death (Morris et al., 2024; MacMahon Copas et al., 2021). At the molecular level, α-syn aggregation is thought to arise from multiple cellular abnormalities. Extensive evidence indicates that PD-related neurodegeneration involves neuroinflammation, oxidative stress, mitochondrial dysfunction, and impaired lysosomal and endosomal pathways. These pathological processes interact and reinforce one another, forming a self-perpetuating cycle that drives progressive neuronal loss (MacMahon Copas et al., 2021; Tolosa et al., 2021). Genetic studies have provided important insights into the pathogenic mechanisms of PD. To date, several causative and susceptibility genes have been identified, including *SNCA, LRRK2, GBA, PRKN, PINK1, and DJ-1* (Tolosa et al., 2021; Day and Mullin, 2021; Bao et al., 2025). Variations in these genes contribute to heterogeneity in molecular pathways and clinical phenotypes. For example, mutations in *SNCA* directly affect α-syn expression and aggregation and are frequently associated with early-onset PD and prominent non-motor manifestations (Gasser, 2023). Mutations in *GBA* impair lysosomal function, thereby promoting abnormal protein accumulation and increasing the risk of cognitive decline (Abeliovich et al., 2021). In contrast, alterations in *PRKN* and *PINK1* disrupt mitochondrial quality control and are associated with relatively selective degeneration of the nigrostriatal system (Bao et al., 2025; Gasser, 2023; Abeliovich et al., 2021; Borsche et al., 2021).

As in AD, CRISPR/Cas9-based genetic screening has become a valuable tool for elucidating the molecular pathology of PD, owing to its high throughput, flexibility, and relatively unbiased design. By systematically knocking out, activating, or repressing genes on a genome-wide scale, this approach enables the identification of key molecular regulators and signaling pathways involved in PD pathogenesis. Together, these advances have improved our understanding of the molecular complexity of PD and have facilitated the identification of potential therapeutic targets and the development of more precise intervention strategies.

### 3.1 CRISPR screening reveals regulators of α-syn degradation and aggregation

**Figure 3:**
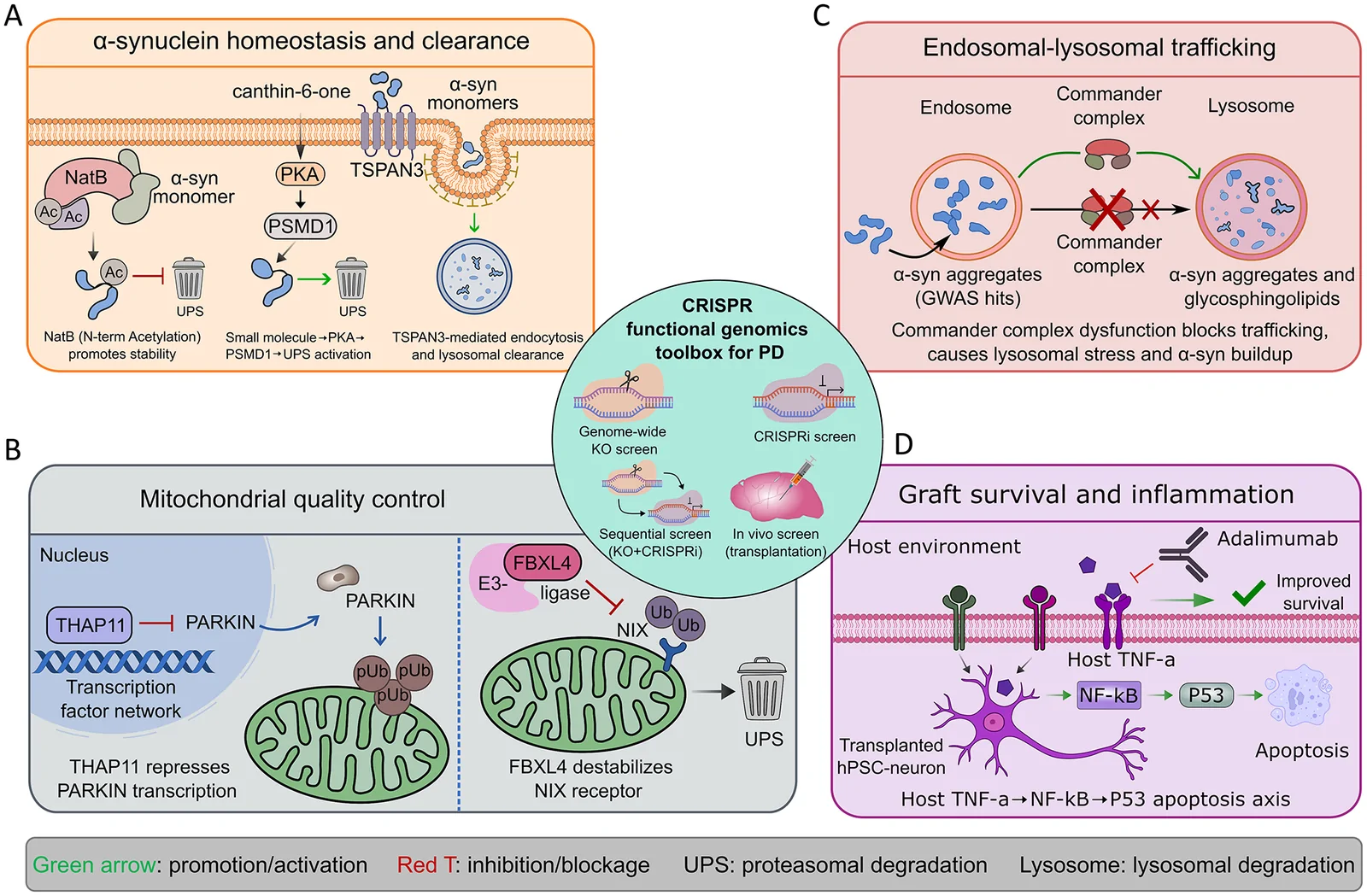
CRISPR functional genomics in PD. **(A)** α-syn homeostasis and clearance. Small molecule canthin-6-one activates the PKA-PSMD1 axis to promote UPS-mediated α-syn degradation. CRISPRi knockdown of NatB reduces N-acetylation, relieving Ube2w inhibition and promoting UPS degradation. TSPAN3 binds α-syn oligomers and promotes their clearance via clathrin/AP2-mediated endocytosis and lysosomal degradation. **(B)** Mitochondrial quality control. *THAP11* transcriptionally represses PARKIN, and FBXL4 promotes degradation of the autophagy receptor NIX, negatively regulating mitophagy. **(C)** Endosomal-lysosomal trafficking. Commander complex dysfunction impairs endosome-to-lysosome trafficking, driving lysosomal stress and α-syn accumulation. **(D)** *In vivo* graft survival and inflammation. *In vivo* CRISPR screen identifies host TNF-α as a driver of NF-κB–p53-mediated apoptosis in transplanted hPSC-DA neurons; pharmacological TNF-α inhibition improves survival.

A genome-wide CRISPR/Cas9 knockout screen investigating α-syn degradation used a dual-fluorescence α-syn reporter system in human HEK293 Tet-On cells, combined with a phenotypic screen of approximately 300 natural compounds (Yuan et al., 2019). This approach identified the indole alkaloid canthin-6-one as a compound that reduced α-syn levels. Subsequent analyses showed that this effect depended on the UPS rather than the autophagy-lysosome pathway (Yuan et al., 2019). A genome-wide CRISPR/Cas9 screen using the GeCKO v2 library further identified *PSMD1* as a mediator of canthin-6-one-induced α-syn degradation (Yuan et al., 2019). Specifically, canthin-6-one was shown to activate the UPS through PKA-dependent upregulation of *PSMD1*, thereby accelerating α-syn clearance (Figure 3A) (Yuan et al., 2019). Together, these findings illustrated the use of CRISPR screening to investigate the mechanism of action of a compound affecting α-syn clearance in a reporter cell model.

A subsequent genome-wide CRISPR/Cas9 loss-of-function screen combined with a bimolecular fluorescence complementation (BiFC) imaging system in human HEK293 cells enabled the identification of regulators of α-syn oligomerization (Hu et al., 2023). This screening strategy identified that tetraspanin 3 (*TSPAN3*) functions as a negative regulator of α-syn aggregation, whereas its homolog *TSPAN7* lacks this activity (Hu et al., 2023). Mechanistic analyses indicated that *TSPAN3* directly interacts with α-syn oligomers, modulates their plasma membrane localization, and promotes their clearance through a clathrin-AP2-mediated endocytic and endolysosomal degradation pathway (Figure 3A) (Hu et al., 2023). These findings highlight *TSPAN3* as a potential molecular target for therapeutic strategies aimed at enhancing α-syn clearance in PD.

Sequential CRISPR screening strategies have also been applied to systematically identify regulators of α-syn expression and stability in human cell models (Santhosh Kumar et al., 2024). In this context, a genome-wide CRISPR/Cas9 KO screen was performed in SKMEL30 human melanoma cells carrying an endogenously tagged *SNCA* locus, followed by a secondary CRISPRi screen in Ngn2-induced human iPSC-derived glutamatergic neurons to reduce the effects associated with complete loss of essential genes. This combined “KO + CRISPRi” approach substantially improved both the coverage and robustness of the screening (Santhosh Kumar et al., 2024). This study revealed that the amino-terminal acetyltransferase B (NatB) acts as a critical positive regulator of α-syn stability by catalyzing its N-terminal acetylation, thereby preventing degradation via the UPS (Santhosh Kumar et al., 2024). Inhibition of NatB activity, either through genetic disruption or partial pharmacological suppression, significantly reduced α-syn protein levels in a manner dependent on the E2 ubiquitin-conjugating enzyme Ube2w (Santhosh Kumar et al., 2024). Importantly, this NatB-α-syn regulatory axis was further validated in human iPSC-derived neurons, confirming its relevance in a human neuronal context (Figure 3A).

### 3.2 CRISPR-based identification of regulators of mitochondrial quality control in PD

In familial PD, mutations in the *PARKIN* gene impair mitophagy, leading to the accumulation of damaged mitochondria in neurons (Potting et al., 2018). Despite its importance, the physiological mechanisms regulating PARKIN protein abundance have remained poorly understood. To address this question, a cellular system enabling monitoring of endogenous *PARKIN* levels was established and combined with a genome-wide CRISPR/Cas9 knockout screen to identify genetic modulators of *PARKIN* abundance (Potting et al., 2018). This screening approach identified 53 positive and negative regulators of *PARKIN* expression, among which a transcriptional repressor network containing *THAP11* emerged as a major regulatory mechanism. Transcriptomic analyses demonstrated that the *PARKIN* locus is a direct target of *THAP11*, and CRISPR-mediated disruption of *THAP11* resulted in increased *PARKIN* expression and enhanced accumulation of phosphorylated ubiquitin, indicative of amplified mitophagy signaling (Figure 3B) (Potting et al., 2018). These regulatory relationships were further validated in human iPSC-derived neurons, underscoring their physiological relevance.

While these studies focused on the classical PINK1/PRKN-dependent mitophagy pathway, the regulatory mechanisms underlying basal mitophagy remains poorly defined. A CRISPR/Cas9 screen targeting the human E3 ubiquitin ligase family and using the mitochondrially targeted monomeric Keima (mt-mKeima) reporter enabled the systematic identification of regulators of basal mitophagy (Elcocks et al., 2023). This screen identified *FBXL4* as a prominent negative regulator of basal mitophagy. Loss of *FBXL4* increased protein levels of the mitophagy receptor NIX (BNIP3L), thereby enhancing mitophagy under steady-state conditions (Elcocks et al., 2023). Mechanistically, *FBXL4* promotes proteasomal degradation of NIX, restraining basal mitophagy (Figure 3B) (Elcocks et al., 2023). Although these experiments were not performed directly in PD models, the screening strategy provides a valuable framework for characterizing mitochondrial quality control pathways relevant to PD pathogenesis.

### 3.3 Lysosomal trafficking as a convergent pathway in PD genetic risk

Beyond classical pathogenic pathways, a recent genome-wide CRISPRi screen provides compelling evidence that genetic risk for PD is associated at the cellular level on defects in lysosomal trafficking (Minakaki et al., 2025). By systematically screening for modifiers of lysosomal glucocerebrosidase (GCase) activity, this work identified the Commander complex, an evolutionarily conserved regulator of endosomal recycling and cargo sorting, as a critical determinant of lysosomal homeostasis in human neurons. CRISPRi-mediated disruption of Commander subunits impaired endosome-to-lysosome transport, reduced lysosomal delivery of GCase and associated proteolytic machinery, and compromised autophagic clearance, leading to the accumulation of α-syn and glycosphingolipids (Minakaki et al., 2025). Integration of functional screening with human genetic data further revealed that multiple Commander components colocalize with PD-associated GWAS loci, supporting this complex as a previously unrecognized genetic risk related pathway (Minakaki et al., 2025). Consistent with these findings, loss of Commander function in human iPSC-derived dopaminergic neurons increased neuronal vulnerability, establishing a causal link between lysosomal trafficking defects and neurodegeneration and highlighting endosome-lysosome membrane dynamics as a central axis in PD pathogenesis (Figure 3C) (Minakaki et al., 2025).

### 3.4 Intrinsic barriers to graft survival revealed by *in vivo* CRISPR screening

While most CRISPR screening studies have been conducted *in vitro, in vivo* CRISPR/Cas9 screening has recently been applied to hPSC-derived dopaminergic neuron transplantation models to systematically investigate intrinsic determinants of graft survival (Kim et al., 2024). Using a pooled CRISPR loss of function screening approach, p53-mediated apoptosis was identified as a major intrinsic factor driving post-transplantation cell loss (Kim et al., 2024). Further mechanistic analyses demonstrated that host-derived inflammatory signaling through tumor necrosis factor-α (TNFα) activates the NF-κB-p53 axis, thereby promoting dopaminergic neuron death and defining a critical TNF-NF-κB-p53 apoptotic pathway (Kim et al., 2024). Importantly, pharmacological inhibition of TNFα signaling, including treatment with the clinically approved TNF inhibitor adalimumab, significantly improved graft survival and functional recovery (Kim et al., 2024). Together, these findings establish a mechanistic framework linking inflammatory stress to intrinsic apoptotic vulnerability in transplanted neurons and highlight a clinically translatable strategy for enhancing the efficacy of cell replacement therapies in PD (Figure 3D).

In summary, these studies illustrate the applicability of CRISPR screening approaches in PD research and their increasing use in the field (Table 2). Across diverse biological processes, including α-syn aggregation, mitochondrial quality control, lysosomal trafficking, and the survival of transplanted neurons, CRISPR-based approaches have enabled detailed analysis of disease relevant molecular mechanisms and facilitated the identification of candidate therapeutic targets. Importantly, the extension of CRISPR screening into stem-cell-derived systems and *in vivo* models has begun to narrow the gap between mechanistic insight and translational relevance, providing a framework for linking genetic perturbations to disease phenotypes in physiologically relevant contexts. Together, these advances highlight the potential of CRISPR screening to contribute to the development of mechanism-informed therapeutic strategies for PD.

**Table 2:** Summary of CRISPR-based functional screens in PD.

Model	Screening type	sgRNA library	Key findings	Reference
HEK293 Tet-On mCherry-α-Syn-EGFP cells	Genome-wide CRISPR/Cas9 knockout screen	GeCKO v2.0 library: 122,417 sgRNAs targeting 19,050 genes	Canthin-6-one promotes α-syn degradation via the UPS; CRISPR screen identified *PSMD1* (RPN2) as the key mediator, acting through PKA-dependent UPS activation.	Yuan et al., 2019
HEK293 cells stably expressing α-syn BiFC-GFP	Genome-wide CRISPR/Cas9 knockout screen	GeCKO V2 human library A and B, containing 112,417 sgRNAs targeting 19,052 genes	Identified *TSPAN3* as a critical negative regulator of α-syn oligomerization; *TSPAN3* interacts with α-syn oligomers and promotes their clearance via clathrin-AP2-mediated endocytosis and lysosomal degradation.	Hu et al., 2023
Human SKMEL30 melanoma SNCA-tagged cell line and iPSC-derived neurons (iNeurons)	Sequential CRISPR screen (CRISPR KO + CRISPRi)	Genome-wide Brunello sgRNA library: 4 sgRNAs per gene, 76,441 sgRNAs targeting 19,114 genes, 1000 NTC sgRNAs	Systematically identified the α-syn regulatory network, found NAA25 as a key enzyme maintaining α-syn N-terminal acetylation, and showed that inhibition of MetAP2 reduces α-syn levels.	Santhosh Kumar et al., 2024
EndoGFP-PARKIN JumpIN TI 293 Cas9+ cells and SH-SY5Y Cas9+ neurons	Genome-wide CRISPR/Cas9 knockout screen (FACS-based sorting of GFP-high/GFP-low)	Genome-wide pooled library: targeting 18,360 protein-coding genes	*THAP11* negatively regulates *PARKIN*; its knockout upregulates PARKIN and enhances mitophagy signaling.	Potting et al., 2018
RPE1-Cas9i cells expressing mt-mKeima	CRISPR/Cas9 knockout screen	Lentiviral sgRNA library containing 2,667 sgRNAs targeting 606 E3 ubiquitin ligase genes 4 sgRNAs per gene) plus 243 NTC sgRNAs	Identified *FBXL4* and *VHL* as negative regulators of basal mitophagy; *FBXL4* loss increases NIX (BNIP3L) protein stability and enhances mitophagy.	Elcocks et al., 2023
dCas9-KRAB HEK-293 cells	Genome-wide CRISPRi screen	lentiviral hCRISPRi-v2 library: 104,535 sgRNAs targeting 18,905 genes	Commander complex emerges as a regulator of lysosomal function and is implicated in PD risk (GBA1 context).	Minakaki et al., 2025
hPSC-derived postmitotic dopamine neuron grafts in NSG mouse striatum	*In vivo* pooled CRISPR kockout screen	Custom pooled sgRNA library: 550 sgRNAs targeting 150 cell-death-related genes, 3 sgRNAs per gene, 100 NTC sgRNAs.	Identified the TNF-NF-κB-p53 axis as a major driver of post-transplant dopamine neuron death; TNFα inhibition (adalimumab) improves graft survival and functional recovery.	Kim et al., 2024

## 4 Applications of CRISPR-based screening in ALS

ALS is a rare but highly fatal neurodegenerative disease characterized by the progressive degeneration of upper and lower motor neurons, leading to muscle weakness, atrophy, and ultimately respiratory failure (Feldman et al., 2022). Epidemiological studies indicate that, although the overall incidence and prevalence of ALS are relatively low, there is substantial geographic variability, with higher rates generally reported in developed regions and a modest male predominance (Feldman et al., 2022; Mehta et al., 2025; Al-Khayri et al., 2024). With population aging and improvements in diagnostic capabilities, the reported incidence of ALS has increased in recent decades (Al-Khayri et al., 2024). Approximately 5–10% of ALS cases are familial (fALS) and are commonly associated with pathogenic variants in genes such as *C9orf72, SOD1, TARDBP*, and *FUS*, whereas the majority of cases are sporadic (sALS) and are thought to arise from complex interactions between genetic susceptibility and environmental factors (Al-Khayri et al., 2024; Riva et al., 2024). Although ALS is relatively rare, it places a considerable burden on affected individuals, their families, healthcare systems, and society, and the observed geographic differences in disease burden merit further investigation (Wolfson et al., 2023).

A major pathological hallmark of ALS is the nucleocytoplasmic mislocalization and aggregation of TAR DNA-binding protein 43 (TDP-43). Aberrant phosphorylation and ubiquitination of TDP-43 disrupt RNA processing and protein homeostasis, ultimately leading to neuronal death (Chong and Souayah, 2025; Ilieva et al., 2023). In a subset of ALS cases, cytoplasmic inclusions containing SOD1 or FUS proteins can also be detected (Ilieva et al., 2023). ALS pathogenesis is driven by multiple, highly interconnected molecular pathways, including dysregulated RNA metabolism, protein aggregation and stress granule dynamics, mitochondrial dysfunction and oxidative stress, microglia- and astrocyte-mediated neuroinflammation, and impairments in axonal transport and synaptic function (Ilieva et al., 2023; Rummens and Da Cruz, 2025; Cunha-Oliveira et al., 2024; Ms et al., 2025). Given that ALS is characterized by numerous overlapping molecular mechanisms, no single gene or pathway can fully explain its complex pathology. CRISPR-based screening technologies enable an in-depth exploration of the genetic networks underlying ALS pathogenesis.

### 4.1 DPR toxicity and intracellular trafficking

**Figure 4:**
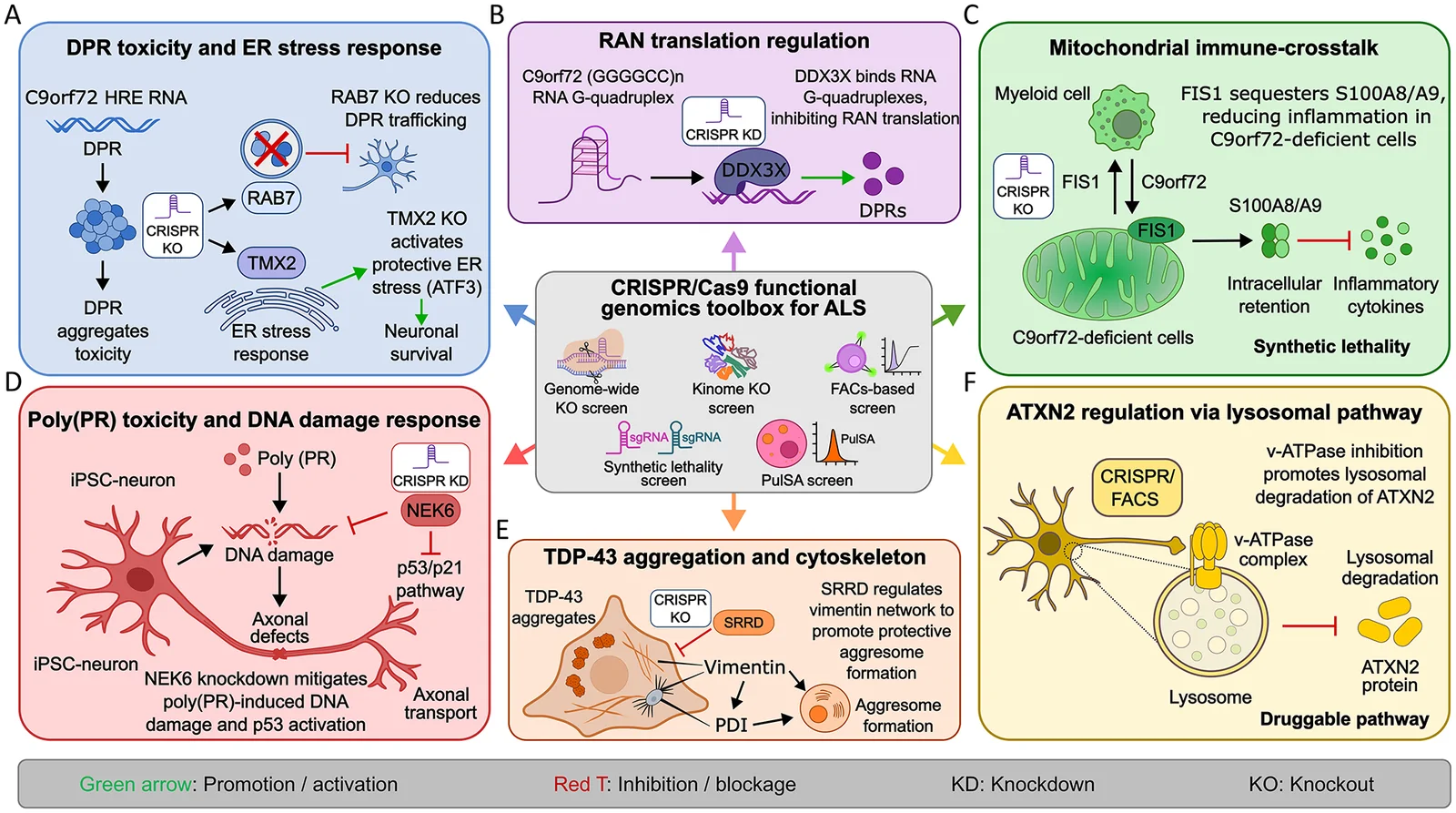
CRISPR functional genomics reveals key pathological mechanisms in ALS. **(A)** DPR toxicity and ER stress response. Loss of *RAB7* impairs DPR endocytosis and intracellular trafficking, resulting in enhanced neuronal damage, whereas loss of *TMX2* upregulates *ATF3* expression and alleviates DPR-induced proteotoxicity. **(B)** Regulation of RAN translation. *DDX3X* suppresses repeat-associated non-AUG (RAN) translation from expanded *C9orf72* repeats, leading to reduced DPR production and attenuated neuronal toxicity. **(C)** Mitochondrial immune-crosstalk. Combined loss of *C9orf72* and *FIS1* disrupts immune homeostasis and leads to synthetic lethality, indicating a compensatory anti-inflammatory function of *FIS1* in the setting of *C9orf72* deficiency. **(D)** Poly(PR) toxicity and DNA damage response. Loss of *NEK6* reduces activation of the p53/p21 DNA damage response pathway, thereby mitigating poly(PR)-induced neuronal toxicity. **(E)** TDP-43 aggregation and cytoskeleton. *SRRD* loss disrupts the vimentin intermediate filament network, thereby limiting aggresome formation and TDP-43 inclusion accumulation. **(F)** *ATXN2* regulation via the lysosomal pathway. Inhibition of the v-ATPase, either genetically or pharmacologically (e.g., etidronate), lowers ATXN2 protein levels and reduces stress granule formation.

In *C9orf72* hexanucleotide repeat expansion associated ALS, dipeptide repeat (DPR) proteins are considered key mediators of neuronal toxicity. To identify molecular mechanisms regulating DPR toxicity, a genome-wide CRISPR/Cas9 knockout screen was performed in Cas9-expressing human K562 cells, identifying multiple pathways linked to DPR induced toxicity, including nucleocytoplasmic transport, RNA processing and splicing, proteasomal degradation, chromatin modification, and ER homeostasis (Kramer et al., 2018). Subsequent validation in primary mouse cortical neurons revealed that knockout of *RAB7* and *TMX2* significantly alleviated DPR induced neuronal damage (Kramer et al., 2018). *RAB7* was found to affect DPR uptake and intracellular trafficking, whereas *TMX2* deficiency mitigated ER stress by upregulating the protective gene *ATF3*, thereby improving neuronal survival. Moreover, reduction of *TMX2* enhanced the survival of C9orf72-ALS patient derived induced motor neurons (iMNs) (Figure 4A) (Kramer et al., 2018). This study provided a systematic analysis of genetic modifiers of C9orf72-associated DPR toxicity, implicated ER stress and intracellular trafficking in the observed phenotypes, and identified *TMX2* as a candidate for further investigation.

### 4.2 Regulation of RAN translation and DPR proteins

In *C9orf72* repeat expansion associated ALS, the expanded hexanucleotide sequence (GGGGCC)n undergoes repeat-associated non-AUG (RAN) translation, generating toxic DPRs that are considered key drivers of neuronal degeneration (Cheng et al., 2019). To identify genes regulating this process, genome-wide CRISPR/Cas9 knockout screens were conducted in Cas9-expressing human RPE1 retinal pigment epithelial cells carrying fluorescent RAN translation reporters (Cheng et al., 2019). This screen revealed that the RNA helicase *DDX3X* directly binds to (GGGGCC)n RNA and suppresses RAN translation through its helicase activity, thereby reducing DPR production. Downregulation of *DDX3X* led to increased DPR accumulation and enhanced toxicity in C9orf72-ALS patient cells and Drosophila models, whereas overexpression of *DDX3X* alleviated nucleocytoplasmic transport defects and improved the survival of patient derived neurons (Cheng et al., 2019). This work uncovered a key regulatory mechanism of RAN translation and identified *DDX3X* as a potential therapeutic target, providing new insights into the molecular pathology and therapeutic strategies for C9orf72-related ALS (Figure 4B).

### 4.3 Mitochondrial-immune regulation in ALS

In C9orf72-associated ALS, a genome-wide synthetic lethal CRISPR/Cas9 screen in human U937 myeloid cells identified FIS1 as a strong genetic interactor of C9orf72 (Chai et al., 2020). Combined loss of *C9orf72* and *FIS1* caused selective lethality in differentiated macrophage-like cells, independent of the canonical role of FIS1 in mitochondrial fission (Chai et al., 2020). Proteomic analyses revealed increased association of FIS1 with S100A8/9 in C9orf72-deficient cells. S100A8/9 are ligands of the receptor for advanced glycation end products (RAGE). These cells also showed reduced extracellular S100A8/9 levels and decreased inflammatory cytokine release, suggesting that intracellular retention of S100A8/9 may attenuate extracellular inflammatory signaling (Chai et al., 2020). These findings suggest a compensatory anti-inflammatory function of *FIS1* in the absence of *C9orf72* and support a potential functional link between mitochondrial homeostasis and immune regulation in ALS pathogenesis (Figure 4C).

### 4.4 DNA damage responses in C9orf72-associated ALS

In C9orf72 hexanucleotide repeat expansion-associated ALS, expanded repeats undergo repeat-associated non-AUG (RAN) translation to generate toxic proline-arginine dipeptide repeat proteins [poly(PR)] that drive neuronal degeneration and axonal dysfunction (Guo et al., 2023). To define genetic regulators of poly(PR) toxicity, an inducible Cas9 expression system was established in human iPSC-derived cortical neurons, followed by a kinome-wide CRISPR/Cas9 knockout screen (Guo et al., 2023). This screen revealed 243 genetic modifiers of poly(PR) toxicity, among which loss of NIMA-related kinase 6 (*NEK6*) most effectively mitigated poly(PR) induced neuronal death and axonal transport defects. Further validation demonstrated that *NEK6* knockdown alleviated mitochondrial axonal transport impairment induced by poly(PR) in iPSC-derived cortical neurons and restored axonal length while reducing aberrant branching in *C9orf72* patient derived neurons and zebrafish models (Guo et al., 2023). Functionally, genetic or pharmacological suppression of *NEK6* reduced the accumulation of DNA damage markers (p53BP1 and γH2AX) and attenuated activation of the p53/p21 signaling pathway, thereby mitigating DNA damage responses triggered by poly(PR) expression or *C9orf72* repeat expansion (Guo et al., 2023). *NEK6* was found to be abnormally upregulated in patient blood cells and brain tissue, suggesting a potential role in disease progression (Figure 4D) (Guo et al., 2023). Together, these findings identify NEK6 as a candidate modifier of poly(PR)-associated DNA damage and axonal phenotypes and support its further investigation as a potential therapeutic target for C9orf72-associated ALS.

### 4.5 TDP-43 aggregation and cytoskeletal organization

In neurodegenerative diseases characterized by impaired proteostasis, misfolded proteins are frequently transported to the perinuclear region to form aggresomes, a process thought to reduce proteotoxic stress and facilitate protein clearance (Sweeney et al., 2024). Traditional genetic screens have largely relied on cell viability or toxicity as readouts, limiting their ability to directly interrogate the molecular mechanisms governing inclusion formation. To overcome this limitation, TDP-43 aggregation was used as a model system in combination with pulse shape analysis (PulSA) and a genome-wide CRISPR/Cas9 knockout screen to identify regulators of protein inclusion formation (Sweeney et al., 2024). This analysis showed multiple known proteostasis related genes, such as *HSPA4* and *UBE3C*, as well as additional regulatory factors, including *METTL5* and *XPO4*. Notably, knockout of *SRRD* significantly reduced the formation of TDP-43 inclusions, establishing *SRRD* as a positive regulator of inclusion formation (Figure 4E) (Sweeney et al., 2024). Building on this observation, the study represents one of the first efforts to directly map the molecular regulatory network controlling inclusion formation through the integration of PulSA-based FACS sorting and genome-wide CRISPR screening. This approach highlighted *SRRD* as a critical regulator of intermediate filament dynamics and aggresome assembly. At the mechanistic level, *SRRD* supports proteostasis and appropriate compartmentalization of misfolded proteins by modulating the vimentin cytoskeletal framework and protein disulfide isomerase (PDI)-mediated disulfide bond formation (Sweeney et al., 2024). Loss of *SRRD* disrupts these processes, thereby impairing the sequestration and subsequent degradation of unfolded proteins (Sweeney et al., 2024). Collectively, these findings support a role for *SRRD* at the interface between cytoskeletal organization and cellular proteostasis and illustrate the use of PulSA-based CRISPR screening to identify regulators of protein inclusion formation.

### 4.6 ATXN2 regulation and therapeutic relevance

In neurodegenerative diseases such as ALS and spinocerebellar ataxia type 2 (*SCA2*), mutations in or overexpression of Ataxin-2 (*ATXN2*) are known to promote neuronal degeneration (Kim et al., 2022). A genome-wide CRISPR/Cas9 knockout screen coupled with FACS analysis was performed in human HeLa cells to map molecular pathways that modulate ATXN2 levels (Kim et al., 2022). Endogenous ATXN2 protein levels were quantified by antibody staining without tagging or overexpression, enabling fluorescence-activated cell sorting of cells with high or low *ATXN2* expression followed by sgRNA enrichment analysis (Kim et al., 2022). Analysis revealed 52 gene knockouts that reduced ATXN2 protein levels, including multiple subunits of the lysosomal vacuolar ATPase (v-ATPase) complex, such as *ATP6V1A* and *ATP6V1C1*. Furthermore, knockdown of v-ATPase subunits markedly decreased ATXN2 protein levels without altering *ATXN2* mRNA expression, indicating post-transcriptional regulation of *ATXN2* homeostasis (Figure 4F).

Several FDA-approved small-molecule inhibitors of v-ATPase, including etidronate, alendronate, and thonzonium, were also shown to reduce *ATXN2* levels in a dose dependent manner in human iPSC-derived neurons and mouse cortical neurons (Kim et al., 2022). Among these compounds, etidronate exhibited no detectable toxicity and was effective following oral administration. In mice, a 7-day oral etidronate treatment reduced brain ATXN2 protein levels by approximately 20% and decreased the formation of stress-induced stress granules (Kim et al., 2022). Overall, v-ATPase represents a druggable pathway for *ATXN2* regulation and underscores the utility of FACS-CRISPR screening for discovering pharmacologically tractable targets relevant to ALS and SCA2.

These studies collectively illustrate the utility of CRISPR-based screening in ALS research (Table 3). From the identification of modifiers of cellular toxicity to the discovery of pharmacologically tractable targets, CRISPR-based approaches have contributed to our understanding of C9orf72-associated pathogenic mechanisms and provided a systematic and scalable framework for identifying and evaluating candidate therapeutic targets.

**Table 3:** Summary of CRISPR-based functional screens in ALS.

Model	Screening type	sgRNA library	Key findings	Reference
Human K562 cells	Genome-wide CRISPR/Cas9 knockout screen	Genome-scale lentiviral sgRNA library: targeting ~20,500 human genes, 10 sgRNAs per gene, ~10,000 NTC sgRNAs	Identified pathways regulating DPR toxicity, including nucleocytoplasmic transport and ER function; *TMX2* reduction protects neurons by alleviating ER stress.	Kramer et al., 2018
Human retinal pigment epithelium (RPE-1) cells	Genome-wide CRISPR/Cas9 knockout screen	Genome-scale lentiviral sgRNA library: targeting 20,567 protein-coding genes, 10 sgRNAs per gene, ~10,000 NTC sgRNAs	Identified *DDX3X* as a repressor of RAN translation; its loss increases DPR levels and toxicity, while overexpression is protective.	Cheng et al., 2019
Human myeloid U937 cells	Sequential CRISPR screening (CRISPR KO + CRISPRi)	Genome-wide lentiviral sgRNA knockout library: 10 sgRNAs per gene,10,000 NTC sgRNAs	Identified *FIS1* as a strong synthetic lethal interactor of C9orf72; loss of both genes caused selective cell death in differentiated macrophage-like cells; *FIS1* compensates for loss of *C9orf72* by binding S100A8/9 and suppressing inflammatory signaling.	Chai et al., 2020
Human iPSC-derived cortical neurons	CRISPR/Cas9 knockout screen	Brunello kinome-wide CRISPR/Cas9 KO library: 3052 sgRNAs targeting 736 kinases; 4 sgRNAs per gene	Identified *NEK6* as a key modifier of poly (PR) toxicity; *NEK6* knockdown or inhibition rescued neuronal death, axonal transport defects, and p53-related DNA damage in *C9orf72*-ALS/FTD models	Guo et al., 2023
Human 293T cells (with TDP-43 aggregation reporter); NGN2-induced neurons for validation	Genome-wide CRISPR/Cas9 knockout screen combined with FACS-based PulSA (Pulse Shape Analysis) to directly quantify TDP-43 inclusion formation	Brunello genome-wide CRISPR sgRNA library: 19,114 sgRNAs targeting 76,441 genes, ~4 sgRNAs per gene, ~1,000 non-targeting controls sgRNAs	Identified canonical proteostasis factors (*HSPA4, UBE3C*) and novel regulators (*METTL5, XPO4, SRRD*); *SRRD* knockout markedly reduced TDP-43 inclusions, indicating its essential role in regulating intermediate filament organization and aggresome assembly	Sweeney et al., 2024
Human HeLa-Cas9 cells (FACS-based endogenous ATXN2 quantification); validated in human iPSC-derived and mouse cortical neurons	Genome-wide CRISPR/Cas9 knockout screen combined with FACS immunostaining for endogenous ATXN2 protein levels	Genome-wide human sgRNA library: 10 sgRNAs per gene, ~21,000 genes, 10,000 NTC sgRNAs	Identified lysosomal v-ATPase complex subunits (e.g., *ATP6V1A, ATP6V1C1*) as positive regulators of ATXN2.	Kim et al., 2022

## 5 Cross-disease integration of CRISPR screening findings

### 5.1 Disease-specific findings and recurring functional themes

**Figure 5:**
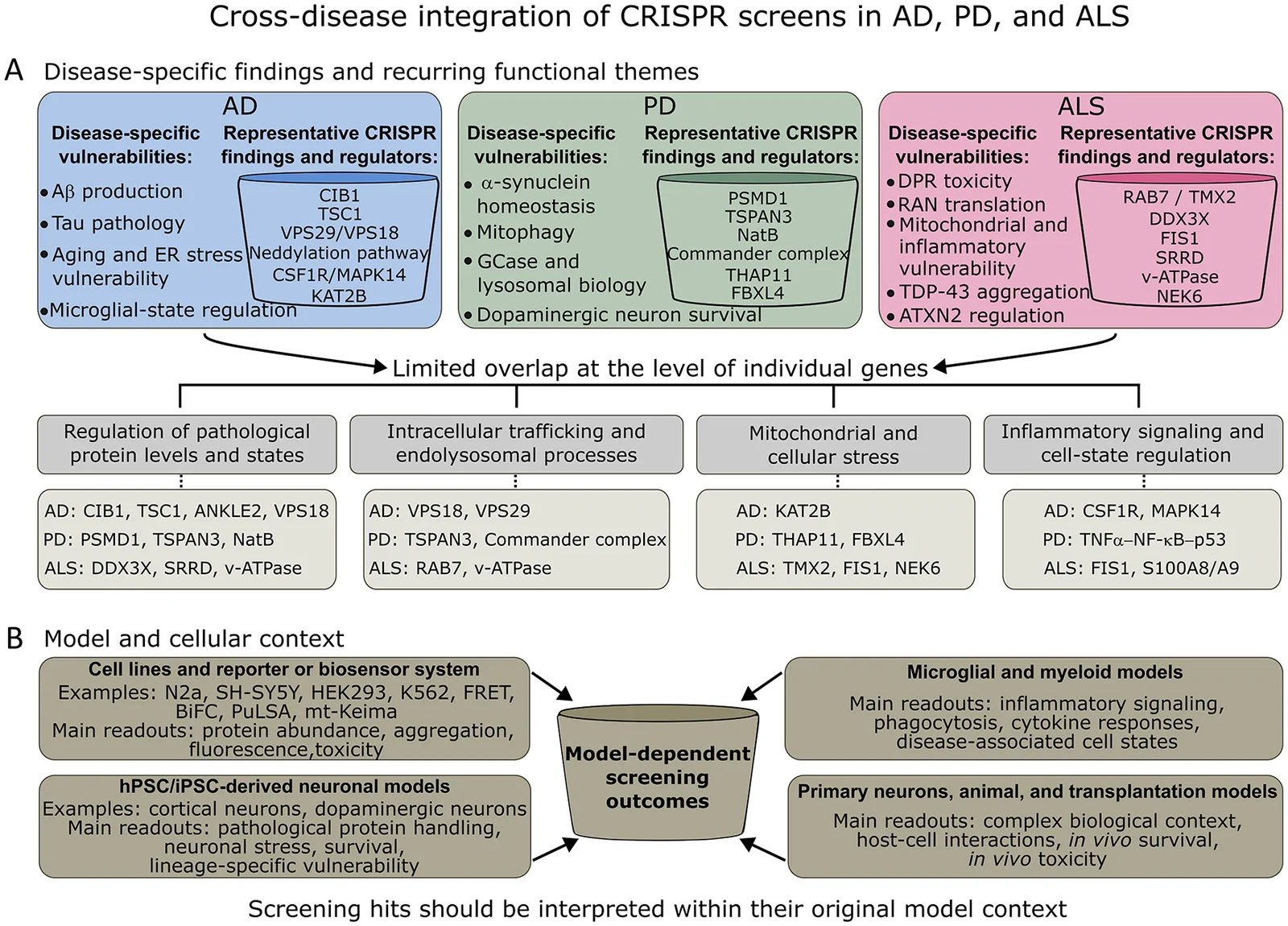
Cross-disease integration of CRISPR screens in AD, PD, and ALS. **(A)** Disease-specific vulnerabilities, representative CRISPR screening findings, and recurring functional themes across AD, PD and ALS. Connections indicate broad functional grouping and do not imply one-to-one relationships or a single shared molecular pathway. **(B)** Experimental models and cell types used in the reviewed studies. Differences in model system, cell type, and phenotypic readout contribute to model-dependent screening outcomes and should be considered when interpreting screening hits. Aβ, amyloid-β; BiFC, bimolecular fluorescence complementation; DPR, dipeptide repeat protein; ER, endoplasmic reticulum; FRET, Foerster resonance energy transfer; GCase, glucocerebrosidase; hPSC, human pluripotent stem cell; iPSC, induced pluripotent stem cell; RAN, repeat-associated non-AUG translation.

CRISPR screens in AD, PD, and ALS show limited overlap at the level of individual genes. This is partly explained by differences in the pathological substrates and phenotypic readouts used in each disease. AD studies have mainly examined Aβ production, Tau abundance, aggregation and propagation, neuronal stress, and microglial states. PD studies have focused on α-syn homeostasis, PARKIN abundance, mitophagy, GCase-related phenotypes, and dopaminergic neuron survival. ALS studies have investigated DPR toxicity, RAN translation, TDP-43 inclusion formation, ATXN2 abundance, and disease-related cellular vulnerability. The identified modifiers therefore largely reflect the specific biological question addressed by each screen (Figure 5A).

Despite this gene-level divergence, several broad functional themes recur across the studies reviewed. One is the regulation of disease-associated protein production, abundance, aggregation, or clearance. Examples include *CIB1* as a regulator of Aβ production; *TSC1* and ubiquitin-proteasome system-related factors as regulators of Tau abundance; and *ANKLE2*, and *VPS18* as modifiers of Tau propagation or aggregation in AD (Chiu et al., 2020; Parra Bravo et al., 2024; Polanco et al., 2023). In PD, *PSMD1, TSPAN3*, and the NatB components were identified in α-syn-related screens (Yuan et al., 2019; Hu et al., 2023; Santhosh Kumar et al., 2024). In ALS, *DDX3X, SRRD*, and v-ATPase-related factors were identified in models of RAN translation, TDP-43 inclusion formation, and ATXN2 abundance (Cheng et al., 2019; Sweeney et al., 2024; Kim et al., 2022). These findings do not establish a single shared protein-quality-control mechanism, but they show that regulation of pathological protein levels and states is repeatedly captured by CRISPR screening.

Intracellular trafficking and endolysosomal-related processes also appear in several disease contexts. In AD, *VPS29* was associated with Tau propagation, whereas *VPS18* was identified as a post-uptake regulator of seeded Tau aggregation (Parra Bravo et al., 2024; Polanco et al., 2023). In PD, *TSPAN3* influenced α-syn oligomer handling, while the Commander complex affected GCase-related and lysosomal phenotypes (Hu et al., 2023; Minakaki et al., 2025). In ALS, *RAB7* modified DPR toxicity, whereas v-ATPase-related factors regulated ATXN2 abundance (Kramer et al., 2018; Kim et al., 2022). These findings indicate partial functional overlap but should not be interpreted as evidence that the identified regulators act through one common pathway.

Mitochondrial regulation and cellular stress represent another recurring but context-dependent functional theme. PD screens identified *THAP11* as a regulator of PARKIN abundance, as well as *FBXL4* and BNIP3L/NIX as regulators of basal mitophagy (Potting et al., 2018; Elcocks et al., 2023). ALS studies implicated *TMX2* in ER stress responses and *NEK6* in poly(PR)-associated DNA damage and axonal phenotypes (Kramer et al., 2018; Guo et al., 2023). The FIS1 finding further linked C9orf72-associated cellular vulnerability to mitochondrial and stress-related processes in a myeloid cell model (Chai et al., 2020). In AD, *KAT2B* was identified as a regulator of tunicamycin-induced ER stress and neuronal apoptosis (Pavlou et al., 2023). These findings suggest that mitochondrial quality control and cellular stress responses recur across disease models, although the specific mechanisms and cellular contexts differ.

Inflammatory signaling and cell-state regulation were mainly detected in microglial, myeloid, or transplantation models. These included CSF1R/MAPK14-associated microglial states in AD (Dräger et al., 2022), TNF-NF-κB-p53 signaling affecting transplanted dopaminergic neuron survival in PD (Kim et al., 2024), and FIS1- and S100A8/9-associated inflammatory responses in ALS-related myeloid cells (Chai et al., 2020). These findings indicate that immune-related screening outcomes are strongly influenced by the cell type and experimental setting. Overall, current evidence supports disease-specific genetic findings with partial overlap at the level of broad cellular functions rather than a unified molecular mechanism across AD, PD, and ALS.

### 5.2 Model and cell type context of CRISPR screening findings

The studies included in this review employed a diverse range of experimental models, including immortalized cell lines, fluorescence-based reporter or biosensor systems, hPSC/iPSC-derived cells, primary neurons, myeloid cells, and *in vivo* or transplantation models. Because these models were designed for different experimental purposes and phenotypic readouts, model context is an important factor when interpreting differences among screening outcomes (Figure 5B).

Immortalized cell lines and biosensor models are well suited for large-scale screening and can provide clear and sensitive phenotypic readouts. For example, N2a and SH-SY5Y cells were used to investigate Aβ production and Tau protein levels, respectively (Sanchez et al., 2021; Chiu et al., 2020); HEK293 reporter systems were used to examine α-syn-related phenotypes (Yuan et al., 2019; Hu et al., 2023); and K562 cells were used to screen for modifiers of C9orf72-associated DPR toxicity (Kramer et al., 2018). Detection systems such as FRET, BiFC, and PulSA further enhanced the identification of Tau or α-syn aggregation and TDP-43 inclusions (Polanco et al., 2023; Hu et al., 2023; Sweeney et al., 2024). These models are therefore particularly suitable for identifying regulators that directly affect specific pathological proteins or predefined phenotypes. hPSC/iPSC-derived cell models provide cellular contexts that more closely resemble the disease-relevant cell types. Cortical neurons have been used to investigate Tau pathology, endoplasmic reticulum stress, and ALS-related toxicity (Saurat et al., 2024; Guo et al., 2023; Kim et al., 2022); dopaminergic neurons have been used to study PD-related neuronal survival; and induced microglia have been used to examine inflammatory and disease-associated cell states. Findings obtained from different neuronal subtypes may also reflect lineage-specific cellular vulnerabilities. Non-neuronal and *in vivo* models provide information that is not captured by isolated neuronal culture systems. Myeloid or macrophage-like models have revealed C9orf72-associated inflammatory responses, whereas dopaminergic neuron transplantation models have demonstrated the influence of host-derived signals on grafted-cell survival. Primary mouse neurons, zebrafish, and mouse models have also been used to evaluate selected screening hits in more complex biological environments.

Screening results should therefore not be compared without considering the model in which they were obtained. Regulators of pathological proteins identified in cell lines or reporter systems may not produce the same phenotypes in disease-relevant neurons, immune cells, or *in vivo* models. Rather than being regarded as directly equivalent, findings from different models should be interpreted as complementary evidence relating to specific pathological substrates, cell types, and experimental endpoints. It is also important to distinguish the strength of validation within an individual study from reproducibility across different models. Validation using independent sgRNAs, genetic rescue, pharmacological intervention, patient-derived cells, or animal models can strengthen the conclusions of a particular study. However, consistent findings across independent studies and different disease-relevant models are required to support the broader disease relevance of a given regulator.

## 6 Discussion

Collectively, CRISPR-based functional genomic screening has become an increasingly important tool for investigating the molecular mechanisms underlying neurodegenerative diseases. By systematically perturbing genes involved in the regulation of distinct pathogenic proteins, existing studies have identified a broad range of genetic modifiers. These efforts have not only improved our understanding of how specific pathogenic proteins are handled at the cellular level, but have also revealed several recurring functional themes, particularly the regulation of pathological protein levels and states, intracellular trafficking, and endolysosomal processes. Post-translational regulatory mechanisms, including neddylation and UFMylation, have also emerged as candidate modulators of proteostasis, pathological protein handling, and neuronal vulnerability, indicating that CRISPR screens can reveal regulatory layers beyond changes in gene expression alone. As summarized in Section 5, these similarities represent partial functional overlap rather than evidence for a single shared molecular pathway across AD, PD, and ALS.

### 6.1 Evidence strength, reproducibility, and limitations of current CRISPR screens

The strength of individual findings varies among studies. Some candidates were supported by mechanistic experiments and validation in complementary models, whereas many others were examined mainly within the original screening system. For example, the involvement of UFMylation in Tau propagation, the Commander complex in GCase-related phenotypes, and NEK6 in poly(PR)-associated stress was supported by additional experimental evidence. However, these findings have not yet been widely reproduced in independent studies. It is therefore important to distinguish validation within an individual study from reproducibility across independent experimental systems. The use of independent sgRNAs, genetic rescue experiments, and complementary perturbation methods can strengthen the evidence for a candidate, but broader relevance requires confirmation in additional disease-relevant models.

Although CRISPR-based functional genomic screening provides a powerful high throughput approach for studying neurodegenerative diseases, current studies still face important limitations in capturing disease complexity (Kampmann, 2020). At the technical level, limitations of CRISPR editing including off-target effects, variability in editing efficiency, and potential Cas9-associated toxicity in vulnerable neurons may introduce noise into screening outcomes, necessitating careful and independent validation of candidate genes (Kampmann, 2020; Ihry et al., 2018; Leibowitz et al., 2021). In addition, CRISPR knockout, CRISPRi, and CRISPRa alter gene function in different ways and to different degrees. Their results are therefore not always directly comparable, particularly for essential genes or genes whose effects depend on the extent of expression change.

Beyond technical issues, a more fundamental limitation arises from the reliance on classical pathological frameworks (Kampmann, 2020). Because high-throughput screens require clear and quantifiable readouts, most studies focus on phenotypes such as protein aggregation or acute cell death (Summers et al., 2024; Okano and Morimoto, 2022). This bias favors the investigation of established hypotheses, including amyloid pathology, Tau and α-syn aggregation, and neuroinflammation (Sengupta and Kayed, 2022; Ali et al., 2025; Mohan Kumar and Talwar, 2025). At the same time, it may reduce the likelihood of identifying regulators that act independently of the selected pathological protein or contribute mainly to slower stages of disease progression. Therefore, the absence of a gene from a particular screen does not necessarily indicate that the gene has no role in disease. It may instead reflect the selected phenotype, model, or observation period.

This reliance on classical frameworks largely reflects a mismatch between commonly used model systems and the nature of neurodegenerative diseases. Neurodegenerative diseases are highly complex and strongly age-related disorders, yet most current screening approaches rely on simplified *in vitro* cell models (Foster-Powell et al., 2025; Ghiasvand et al., 2024; Kolagar et al., 2020). In particular, widely used iPSC-derived cells retain a fetal-like biological age, meaning that many studies effectively use “young” cellular models to study diseases that develop in aged nervous systems (Bohnke et al., 2018; Mertens et al., 2018; Cornacchia and Studer, 2017). As a result, critical features related to aging and systemic context are often not adequately captured (Bohnke et al., 2018; Mertens et al., 2018; Cornacchia and Studer, 2017). Interactions among neurons, glial cells, and the surrounding tissue environment are also difficult to reproduce in simplified cell culture systems. In addition, to generate detectable signals within a limited experimental timeframe, screening strategies frequently rely on high-intensity acute perturbations to model disease processes that in humans evolve over decades. This discrepancy between acute screening conditions and chronic disease progression suggests that some screening hits may reflect acute stress responses or short-term cellular adaptation rather than mechanisms that specifically regulate long term neurodegeneration (Kampmann, 2020).

Immortalized cell lines also have important limitations. Although SH-SY5Y human neuroblastoma cells exhibit some neuronal characteristics, they remain proliferative and do not develop the elaborate neuronal processes and synaptic connections observed in human neurons. In the study by Sanchez et al., primary and secondary screens in SH-SY5Y cells identified and validated multiple regulators of Tau protein levels. However, among 43 selected candidates subsequently tested in Ngn2-induced human excitatory glutamatergic neurons, only five were validated (Sanchez et al., 2021). The authors noted that this low validation rate may reflect intrinsic differences between SH-SY5Y cells and Ngn2-induced neurons, but it may also be influenced by the timing of gene editing during neuronal differentiation. Moreover, the Ngn2-induced neurons themselves did not fully reproduce adult human brain Tau expression, as they lacked the 4R Tau isoform at the examined time point and showed different total Tau levels (Sanchez et al., 2021). This example illustrates that screening outcomes and subsequent validation can be strongly influenced by the characteristics and experimental design of both the initial screening model and the validation model (Sanchez et al., 2021).

Animal models provide tissue context and interactions among different cell types, but species-specific differences may limit the direct translation of their findings to human disease. For example, Mancuso et al. transplanted human stem cell-derived microglia into the brains of AppNL-G-F mice and found that human microglia developed several responses to Aβ pathology that differed from those observed in mouse microglia, including more pronounced HLA-associated and cytokine/chemokine-associated states (Mancuso et al., 2024; Sokratian et al., 2024). In addition, mouse α-syn fibrils were recently shown to differ structurally and functionally from fibrils associated with human Lewy body diseases, with differences in fibril packing, fragmentation sensitivity, immune responses, and pathological spreading (Mancuso et al., 2024; Sokratian et al., 2024). These findings illustrate that animal models may reproduce selected aspects of neurodegenerative pathology but may not fully reflect the corresponding molecular and cellular responses in humans (Mancuso et al., 2024; Sokratian et al., 2024).

Current CRISPR screens reveal limited genetic overlap across AD, PD, and ALS, despite shared pathological features. This divergence likely reflects a target dependent bias inherent to existing screening designs. Screens are typically constructed around specific pathological proteins, which favors the identification of regulators linked to the unique biochemical properties of each substrate (for example, CIB1 in Aβ regulation or NatB in α-syn stability), rather than factors acting upstream across multiple disease contexts (Chiu et al., 2020; Santhosh Kumar et al., 2024). As a result, current approaches have been effective in defining protein specific handling mechanisms but they were not designed primarily to identify regulators shared by different diseases. The limited convergence among studies may therefore result from differences in pathological substrates, cell types, models, perturbation methods, and phenotypic readouts. It may also reflect genuine biological differences among AD, PD, and ALS. Current evidence does not distinguish clearly between these possibilities and does not support the existence of a single dominant regulator of neurodegeneration.

### 6.2 Translational relevance and therapeutic potential

The therapeutic value of a screening hit cannot be determined from a protective genetic phenotype alone. Some targets, such as enzymes, receptors, and extracellular signaling molecules, may be easier to modulate pharmacologically than essential protein complexes or cellular machinery with broad physiological functions (Nelson et al., 2015; Minikel et al., 2024; Razuvayevskaya et al., 2024). In addition, complete gene knockout may produce a different effect from partial or transient pharmacological modulation (Duffy et al., 2025). These differences need to be considered when screening hits are evaluated as potential therapeutic targets. Other factors, including cell-type specificity, delivery to the central nervous system, long-term toxicity, and the disease stage at which treatment is initiated, may also determine whether a candidate can be developed further (Razuvayevskaya et al., 2024; Miller et al., 2022; van Dyck et al., 2023).

Despite these limitations, several studies reviewed here provide examples in which CRISPR screening findings were followed by pharmacological validation. In an *in vivo* PD transplantation model, inhibition of TNF signaling with adalimumab improved the survival of grafted dopaminergic neurons (Kim et al., 2024). Inhibition of KAT2B with L-Moses reduced tunicamycin-induced cell death in cortical and dopaminergic neurons (Pavlou et al., 2023). Partial inhibition of NatB function reduced endogenous α-syn levels in human neurons, whereas pharmacological inhibition of v-ATPase activity reduced ATXN2 abundance in neuronal models and in the mouse brain (Kim et al., 2022). These studies provide preclinical evidence that selected screening findings can be further examined through pharmacological modulation. However, they do not demonstrate clinical efficacy. Further testing in chronic disease models, independent experimental systems, and long-term safety studies will be required before these candidates can be considered for therapeutic development.

### 6.3 Emerging CRISPR screening approaches

Several emerging CRISPR approaches may help address some of the limitations of conventional pooled screens. CROP-seq and Perturb-seq combine defined genetic perturbations with single-cell transcriptomic analysis. They can therefore identify responses that occur only in particular cell states or cellular subpopulations and may be missed by population-averaged readouts (Santinha et al., 2023; Zheng et al., 2024). Among the studies reviewed here, CROP-seq has already been used to examine perturbation-dependent states in induced human microglia (Dräger et al., 2022). Although several proof-of-concept studies have applied single-cell CRISPR approaches in neuronal and glial models, their use remains relatively limited and uneven across AD, PD, and ALS compared with conventional pooled CRISPR screens (Santinha et al., 2023; Zheng et al., 2024; Shen et al., 2026).

CRISPR perturbations can also be combined with transcriptomic, epigenomic, proteomic, imaging, or spatially resolved readouts (Shen et al., 2026; Binan et al., 2025; Saunders et al., 2025). These approaches may provide more information than a single phenotypic endpoint and may help distinguish primary effects of genetic perturbation from secondary stress responses. Base editing and prime editing may further allow disease-associated variants to be studied through precise sequence changes, rather than only through complete gene loss (Belli et al., 2025). Barcoded *in vivo* screening and *in vivo* Perturb-seq may also allow genetic effects to be examined in intact tissues, where interactions among different cell types and signals from the host environment are retained (Santinha et al., 2023; Zheng et al., 2024; Shen et al., 2026; Ramani et al., 2025).

These approaches are still at an early stage in neurodegenerative disease research. Their application remains limited by editing efficiency, delivery, screening scale, cost, and data analysis (Santinha et al., 2023; Belli et al., 2025; Ramani et al., 2025). They are therefore more likely to complement conventional CRISPR knockout, CRISPRi, and CRISPRa screens than to replace them.

### 6.4 Future directions and unresolved challenges

Future progress in CRISPR-based functional genomic screening for neurodegenerative diseases will depend less on increasing screening scale and more on improving experimental design. To better capture disease relevant mechanisms, future studies should move beyond the exclusive use of assays based on pathological protein abundance, aggregation, or acute cell death. Partial genetic perturbations, longer observation periods, and more physiologically relevant stress conditions may better reflect the gradual development of neurodegenerative disease (Kampmann, 2020). Protein centered screens remain useful for identifying mechanisms related to a particular pathological substrate, but they should be combined with readouts that examine broader cellular pathways, stress responses, and changes in cell state over time.

In parallel, the use of advanced model systems, including directly converted neurons, models with induced aging, three dimensional organoids, co-culture systems, and *in vivo* screening platforms, may help incorporate features of aging, intercellular interactions, and tissue context that are largely absent from conventional *in vitro* models (Dawson and Dawson, 2025; Masuda-Suzukake et al., 2025; Zhang et al., 2025; Larriba-González et al., 2025). Coupling these approaches with high dimensional readouts, such as single cell transcriptomics, multiomic analysis, and high-content imaging, may allow screening results to be interpreted beyond isolated phenotypic endpoints (Tian et al., 2019).

Several questions remain unresolved. It is still unclear whether the limited overlap among studies mainly reflects the biological heterogeneity of neurodegenerative diseases or differences in screening design. Many candidate genes have not been tested across matched cell types and models, making it difficult to separate disease specific effects from model-specific effects (Chardon et al., 2024; Li et al., 2025). It is also unknown whether broadly acting upstream regulators are missed because most current screens focus on individual pathological proteins, or whether different neurodegenerative diseases are controlled by largely context dependent regulatory networks.

Addressing these questions will require direct comparison of the same genetic perturbations across disease relevant cell types and models under matched experimental conditions (Chardon et al., 2024). Protein specific readouts could be combined with measurements of cell state, stress responses, and changes over time (Li et al., 2025). Such comparisons may help determine whether common regulatory mechanisms can be identified when differences in screening design are reduced, or whether similar cellular vulnerabilities are controlled by different genes in different disease contexts.

Greater standardization will also be needed to improve comparison and reproducibility across studies. Cell models, perturbation libraries, editing efficiency, phenotypic definitions, and validation criteria should be reported consistently (Yao et al., 2024). Prioritized candidates should be tested in independent laboratories and in more than one disease relevant model. These steps will help distinguish findings that are specific to an individual screening system from those with broader disease relevance and therapeutic potential.
